# Helminth Coinfection Does Not Affect Therapeutic Effect of a DNA Vaccine in Mice Harboring Tuberculosis

**DOI:** 10.1371/journal.pntd.0000700

**Published:** 2010-06-08

**Authors:** Fabiani G. Frantz, Rogério S. Rosada, Camila Peres-Buzalaf, Franciele R. T. Perusso, Vanderlei Rodrigues, Simone G. Ramos, Steven L. Kunkel, Célio L. Silva, Lúcia H. Faccioli

**Affiliations:** 1 Faculdade de Ciências Farmacêuticas de Ribeirão Preto, Universidade de São Paulo, Ribeirão Preto, São Paulo, Brazil; 2 Faculdade de Medicina de Ribeirão Preto, Universidade de São Paulo, Ribeirão Preto, São Paulo, Brazil; 3 Department of Pathology, University of Michigan Medical School, Ann Arbor, Michigan, United States of America; Leiden University Medical Center, Netherlands

## Abstract

**Background:**

Helminthiasis and tuberculosis (TB) coincide geographically and there is much interest in exploring how concurrent worm infections might alter immune responses against bacilli and might necessitate altered therapeutic approaches. A DNA vaccine that codifies heat shock protein Hsp65 from *M. leprae* (DNAhsp65) has been used in therapy during experimental tuberculosis. This study focused on the impact of the co-existence of worms and TB on the therapeutic effects of DNAhsp65.

**Methodology/Principal Findings:**

Mice were infected with *Toxocara canis* or with *Schistosoma mansoni*, followed by coinfection with *M. tuberculosis* and treatment with DNAhsp65. While *T. canis* infection did not increase vulnerability to pulmonary TB, *S. mansoni* enhanced susceptibility to TB as shown by higher numbers of bacteria in the lungs and spleen, which was associated with an increase in Th2 and regulatory cytokines. However, in coinfected mice, the therapeutic effect of DNAhsp65 was not abrogated, as indicated by colony forming units and analysis of histopathological changes. *In vitro* studies indicated that Hsp65-specific IFN-γ production was correlated with vaccine-induced protection in coinfected mice. Moreover, in *S. mansoni*-coinfected mice, DNA treatment inhibited *in vivo* TGF-β and IL-10 production, which could be associated with long-term protection.

**Conclusions/Significance:**

We have demonstrated that the therapeutic effects of DNAhsp65 in experimental TB infection are persistent in the presence of an unrelated Th2 immune response induced by helminth infections.

## Author Summary

From 14 diseases considered by WHO as Neglected Tropical Diseases, four involve helminth infections, such as schistosomiasis and soil-transmitted helminthiasis. Toxocariasis is a soil-transmitted worm highly prevalent in many developing countries, while schistosomiasis causes an annual mortality of 14,000 deaths per year, with 200–300 million infected people and 10% at risk of infection worldwide. Additionally, tuberculosis (TB) remains one of the leading causes of morbidity and mortality in many settings, particularly in the world's poorest countries. Mycobacteria and helminths are co-endemic and induce opposing patterns of immune responses in the host, recognized as Th1 and Th2 respectively. These co-existing patterns could be associated with the failure of TB vaccines. In this sense, we investigated the inflammatory and immune response in a coinfection model with *T. canis* or *S. mansoni* and *M. tuberculosis* analyzing the effects of an immunotherapy that has previously shown efficacy in experimental TB. This immunotherapy is based on a DNA vaccine that codifies a mycobacterial heat shock protein (hsp65), which can prevent TB in a prophylactic and also therapeutic setting. In this work, we show that helminth coinfection does not abrogate the therapeutic effects of DNAhsp65 vaccine against TB.

## Introduction

Helminth parasites cause significant morbidity worldwide, with estimates indicating that approximately one-third of the almost three billion people living on less than two US dollars per day in developing regions of sub-Saharan Africa, Asia, and the Americas are infected with one or more helminths [1], [2]. Helminth infections are potent Th2 response inducers in both humans and experimental models, characterized by eosinophilia, high titers of circulating IgE, enhanced Th2 cytokine profile [*e.g.*, increased secretion of interleukin 4 (IL-4) and IL-5], and regulatory (IL-10, TGF-β) cytokines and reduced Th1 type cytokines [*e.g.*, interferon (IFN)-γ] [3]. Toxocariasis is an underestimated soil-transmitted helminthiasis that primarily affects people in developing countries [4], [5], including Brazil, where prevalence rates reach ∼40% [6], [7], while Schistosomiasis causes 14000 deaths per year, with 200–300 million infected people and 10% at risk of infection worldwide, according to the Global Burden of Disease Study [8]. Additionally, tuberculosis remains one of the leading causes of morbidity and mortality in many settings, particularly in the world's poorest countries. It is estimated that of the approximately 8.9 million people that developed tuberculosis in 2004, nearly 1.7 million people died from it [9].

Studies in animal models of Th1-inducing pathogens and in pre-clinical trials with certain vaccines showed that infection with helminthic parasites impaired Th1 immune responses [10], [11], [12], [13], [14]. This decrease in immunogenicity of BCG (Bacillus Calmete-Guérin, the current vaccine against tuberculosis) was described in a study conducted in a rural community in Ethiopia where healthy or helminth-infected volunteers received anti-helminthic therapy or placebo, and were subsequently vaccinated with BCG. In that study, the authors showed that helminth infection impaired IFN-γ secretion and increased TGF-β production by peripheral blood mononuclear cells stimulated *in vitro* with PPD (purified protein derivative) after BCG vaccination [15]. These findings highlight potential explanations for the apparent failure of BCG to prevent pulmonary tuberculosis in populations inhabiting tropical regions.

Vaccines against tuberculosis (TB) under development include attenuated or enhanced live whole-cell, inactivated whole-cell, subunit, virus-vectored, and DNA vaccines followed by several immunization strategies using prime-boost protocols. Several of these candidate vaccines have demonstrated activity in animal models that is equal or superior to that of BCG; trials in human subjects are currently under way [16]. We have previously demonstrated that a DNA vaccine encoding the mycobacterial 65-kDa heat shock protein (DNAhsp65) protected mice and guinea pigs from challenge with a virulent strain of *Mycobacterium tuberculosis* (Mtb) [17], [18] and, further, cured previously infected mice when administered as naked DNA by intramuscular injection [19]. We have also shown that therapeutic use of DNAhsp65 in combination with anti-mycobacterial drugs shortens the duration of TB treatment, improves treatment of latent TB infection, and is effective against multi-drug resistant TB [20]. However, to improve therapies and vaccination protocols against tuberculosis it is important to investigate the influence of helminth coinfection on the immune response during TB [21] and its effects on treatment and immunization. We previously showed that infecting BALB/c mice with the helminth *Toxocara canis* elicited a Th2 response but did not alter susceptibility to subsequent infection with *M. tuberculosis* [22]. In contrast, *Schistosoma mansoni* infection can strongly enhance susceptibility to TB [13] and impair the protective effects of BCG vaccination [23].

Here, we show that the immune and pathological responses induced by coinfection with *T. canis* or *S. mansoni* and TB did not abrogate the therapeutic effect of the DNAhsp65 vaccine. We found that the therapeutic effect was maintained due to Hsp65-specific IFN-γ production as well as an inhibition of TGF-β production in the lung. These results suggest that the DNAhsp65 vaccine may provide an elegant vaccination strategy in tropical countries where infections with multiple pathogens are common and lead to altered immune responses.

## Materials and Methods

### Animals

Specific pathogen-free female 6-week-old BALB/c mice were obtained from the animal facilities of Faculdade de Ciências Farmacêuticas - Universidade de São Paulo and bred in a SPF facility. All experiments were approved and conducted in accordance with the guidelines of the Animal Care Committee of the University. Infected animals were housed in cages within a laminar flow safety enclosure and kept in a Biosafety Level 3 biohazard animal room.

### Plasmid construction and purification

DNAhsp65 vaccine was derived from the pVAX1 vector (Invitrogen, Carlsbad, CA, USA), which had previously been digested with *BamHI* and *Not I* (Invitrogen) and a 3.3-kb fragment (corresponding to the *M. leprae* HSP65 gene) was inserted. The vector pVAX1 was used as control. Plasmids were replicated in DH5α *Escherichia coli* and purified with Endofree Plasmid Giga kit (Qiagen, Valencia, CA, USA) according to the manufacturer's protocol. Endotoxin levels were determined using a QCL-1000 Limulus amebocyte lysate kit (Cambrex Company, Walkersville, MD, USA) and were less than 0.1 endotoxin units (EU)/µg DNA.

### Experimental infection

#### *T. canis*

*T. canis* eggs were obtained by the Olson and Schulz method [24], modified by Faccioli *et al.* [25]. In brief, pregnant female worms were recovered from dogs. Then, the eggs were rescued from the worm uterus, washed and maintained in 0.5% formalin at 37°C in shallow dishes, where they developed into an infective stage. Before being used, the eggs were thoroughly washed with saline. Infective doses of 500 embryonated eggs in 0.5 ml of saline were prepared. Mice were infected by gastric intubation via a metal cannula. Control animals received only 0.5 ml of saline.

#### *S. mansoni*

*S. mansoni* LE strain was maintained by routine passage through Biomphalaria glabrata snails and BALB/c mice. Infected snails were induced to shed cercariae under light exposure for 2 h; cercariae were recovered by sedimentation on ice. The number of cercariae in suspension was determined and mice were percutaneously infected with 30 cercariae/mouse using a procedure adapted from the ring method [26].

#### *M. tuberculosis*

The H37Rv strain of *M. tuberculosis* (American Type Culture Collection, Rockville, MD) was grown in 7H9 Middlebrook broth (Difco Laboratories, Detroit, MI) for 7 days. The culture was harvested by centrifugation and the cell pellet was resuspended in sterile phosphate buffered saline (PBS) and vigorously agitated. The homogeneous suspension was filtered through a 2-µm filter (Millipore, Bedford, MA). Because CFU determination takes 4 to 6 weeks, we used fluorescein diacetate (Sigma, Saint Louis, MO) and ethidium bromide staining [27] to rapidly assess the viability of *M. tuberculosis* cultures upon infection. An anterior midline incision was made to expose the trachea. A 30-gauge needle attached to a tuberculin syringe was inserted into the trachea and intratracheal dispersion was used to introduce 10^4^ CFU (in *S. mansoni* coinfection experiments) or 10^5^ (in *T. canis* coinfection experiments) viable CFU of *M. tuberculosis* H37Rv in 100 µl of PBS into the lungs [28]. Control mice received only intratracheal PBS.

As shown on the chart in Figure S1, infection with *M. tuberculosis* was performed 18 days or 42 days after the *Toxocara canis* or *S. mansoni* infection, respectively. At these two time-points, each worm induces an optimal Th2 response [29], [30] due to the visceral larva migrans syndrome present in toxocariasis and the incidence of eggs in the liver and intestine of *S. mansoni* infected hosts.

### DNA vaccination therapy

**Figure 1:**
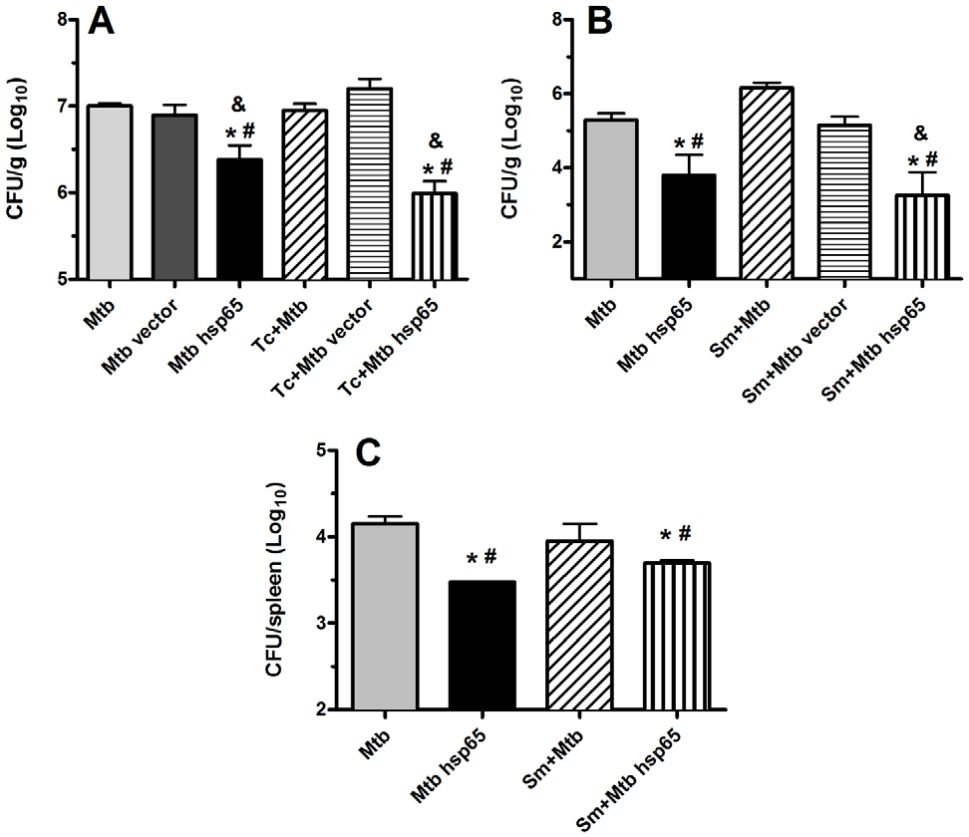
Number of *M. tuberculosis* colony forming units (CFU) recovered from coinfected mice. Protective efficacy was assessed by replication of TB bacilli in lungs (A, B) and spleen (C) at the end of DNAhsp65 therapy in animals coinfected with *T. canis* (A) or *S. mansoni* (B, C). Data represent the mean log_10_ CFU counts ± SD of five mice per group from one of three independent experiments. *p<0.05 versus Mtb; ^#^p<0.05 versus Sm+Mtb or Tc+Mtb; ^&^p<0.05 versus respective vector group.

DNA vaccination was initiated 30 days after TB induction by injection of 50 µg of plasmid DNA in 50 µl of saline into the quadriceps muscle of each hind leg, on four occasions at 10-day intervals. Ten days after the last dose, mice were killed and bacterial growth, lung histology, and cytokine production by lung or spleen cells were accessed (Figure 1). All figures represent the results from one of three representative experiments: first lungs were removed, using the right cranial lobe and right middle lobe for homogenization, the right caudal lobe for fixation and the left lobe and accessory lobe for CFU. The spleen was also removed from the same mice.

### Determination of *M. tuberculosis* colony forming units (CFU) in lungs

Recovery of *M. tuberculosis* was performed as described previously [31]. Briefly, the number of live bacteria recovered from the lungs was determined as CFU by plating 10-fold serial dilutions of homogenized tissue on Middlebrook 7H11 agar (Difco) incubated at 37°C. Colonies were counted after 28 days. Results are expressed as log_10_ of CFU/g lung.

### Measurement of cytokines and nitrite levels in lung tissues

For cytokine measurements, lungs were removed and homogenized in 2 ml RPMI 1640, centrifuged at 450×*g* and the supernatant was stored at −70°C until assayed. Commercially available enzyme-linked immunosorbent assay (ELISA) antibodies were used to measure IL-4, IL-5, IL-10, TGF-β, IL-12, and IFN-γ (OptEIA™ BD-Pharmingen, San Diego, CA). Plates were coated with 100 µl/well of the capture antibody (1–4 µg/ml) diluted in Coating Buffer (0.1 M Sodium Carbonate, pH 9.5) and incubated overnight at 4°C. Plates were washed 5 times with 300 µl/well Wash Buffer (PBS with 0.05% Tween-20) and non-specific binding was blocked by addition of 200 µl/well Assay Diluent (PBS with 10% FBS, pH 7.0), and incubated at room temperature for 1 hour. After washing as above, 100 µl of standards, samples, and controls were added into appropriate wells and incubated for 2 hours at RT. The plates were washed as above and 100 µl of Working Detector [Detection Antibody (0.5–2.0 µg/ml)+SAv-HRP reagent] was added to each well for 1 hour at room temperature. After 7 additional washes, 100 µl of Substrate Solution (BD Pharmingen™ TMB SubstrateReagent Set) was added to each well and incubated for 30 minutes at room temperature in the dark. The reaction was stopped by adding 50 µl of Stop Solution (2 N H_2_SO_4_) to each well. Optical density was measured at 450 nm within 30 minutes of stopping the reaction.

Nitric oxide (NO) production was assessed by measuring nitrite levels in lung homogenates using the Greiss reagent method [32]. Data are presented as micromoles of NO_2_^−^.

### Spleen and lung cell cultures and cytokine determination

Spleens from mice killed 10 days after the last DNA dose were aseptically removed and minced and the released cells were washed three times in RPMI 1640 (Gibco BRL, Grand Island, USA). Minced lungs were next treated with 5 ml/lung of collagenase solution (0.1 g of Type IV collagenase [Sigma Chemical Co., St. Louis, USA] in 45 ml of RPMI 1640) and allowed to shake for 30 minutes at 37°C. The suspension was centrifuged at 450×*g* for 10 minutes and the pellet was suspended in RPMI supplemented with 10% fetal bovine serum (Gibco BRL), penicillin (100 U/ml, Gibco BRL), streptomycin (100 µg/ml, Gibco BRL). Cells from lung or spleen were suspended at 5×10^6^ cells per ml in supplemented RPMI and dispensed into 96-well flat-bottom microtiter plates in a volume of 0.1 ml. A total of 10 mg/ml recombinant Hsp65 was added to wells (0.1 ml) in triplicate and maintained for 48 h at 37°C. Commercially available ELISA antibodies were used to measure IFN-γ (OptEIA™ BD-Pharmingen) in supernatants of cultured cells as above.

### Statistical analysis

Data are represented as mean ± SEM, n = 5 (PBS group) or n = 6 (other groups) and were analyzed with using GraphPad Prism version 4.02 for Windows (GraphPad Software, San Diego, CA). Comparisons were performed using unpaired *t* tests for CFU analyses or one-way ANOVA with Bonferroni's post test for other experiments. Differences were considered significant if *P*<0.05. Experiments were repeated 2–3 times and similar results were observed in all experiments.

## Results

### DNAhsp65 therapy in helminth-TB coinfected mice causes decrease of *M. tuberculosis* replication and sustains a preserved lung architecture

As shown previously [19], [28], DNAhsp65-therapy was effective in reducing the number of CFU in Mtb-infected mice (black bars, Figure 1A–C), whereas the treatment with empty vector was not. We have also demonstrated previously that coinfection with *T. canis* could not increase susceptibility to TB [22]. Here, we confirmed this data (Figure 1A), showing that numbers of bacteria in the lung of *T. canis*-coinfected mice were quite similar to those in Mtb-infected mice. On the other hand, *S. mansoni-*coinfection resulted in an increase in CFU counts, demonstrating greater susceptibility to *M. tuberculosis* bacilli under these conditions, confirming previously published data [13].

To evaluate whether helminth infection could impair DNAhsp65-induced protection against *M. tuberculosis*, mice were coinfected with worms and treated with DNAhsp65 following the time line presented in Figure S1. Although *T. canis*-coinfection did not change bacterial burden in the lungs, we also observed that this helminth did not influence the therapeutic effect of DNAhsp65 (Figure 1A), since we observed a similar reduction in CFU in lungs tissue in both Mtb hsp65 and Tc+Mtb hsp65 groups. Surprisingly, therapy with DNAhsp65 was also highly effective in *S. mansoni-*coinfected animals, as CFUs were reduced in lungs of this group (Figure 1B). Due to the ability of *S. mansoni* to alter the immune response in a concomitant Mtb infection [13], different from *T canis* that does not influence in bacteria burden [22], we also evaluated CFU levels in the spleen after treatment with DNAhsp65 and found that the differences in CFU counts suggested that the DNAhsp65-therapeutic effect observed in lungs is maintained systemically (Figure 1C).

Histological analysis of the lungs also revealed that cellular accumulation, cellular organization and pulmonary parenchyma commitment were similar in *T. canis* coinfected and *M. tuberculosis* infected mice. In contrast, *S. mansoni* coinfection induced greater lung injury when compared to *M. tuberculosis* infection itself resulting in increased inflammatory cells numbers and greater tissue congestion. Treatment with empty vector did not alter lung pathology or CFU either in mice infected with *M. tuberculosis* or coinfected with helminths. In contrast, DNAhsp65 therapy resulted in a significant decrease in lesions in the Mtb group and the coinfected groups. Histological sections of HE-stained lungs from coinfected and *M. tuberculosis* infected mice were characterized predominantly by the presence of macrophages and lymphocytes that accumulated mainly in perivascular and peribronchial areas. Immunotherapy with DNAhsp65 partially reversed the pathologic effects resulting in a large preserved area in the lung parenchyma (Data not shown).

### The therapeutic effect of DNAhsp65 was maintained during coinfection due to modulation of immune response in lungs

**Figure 2:**
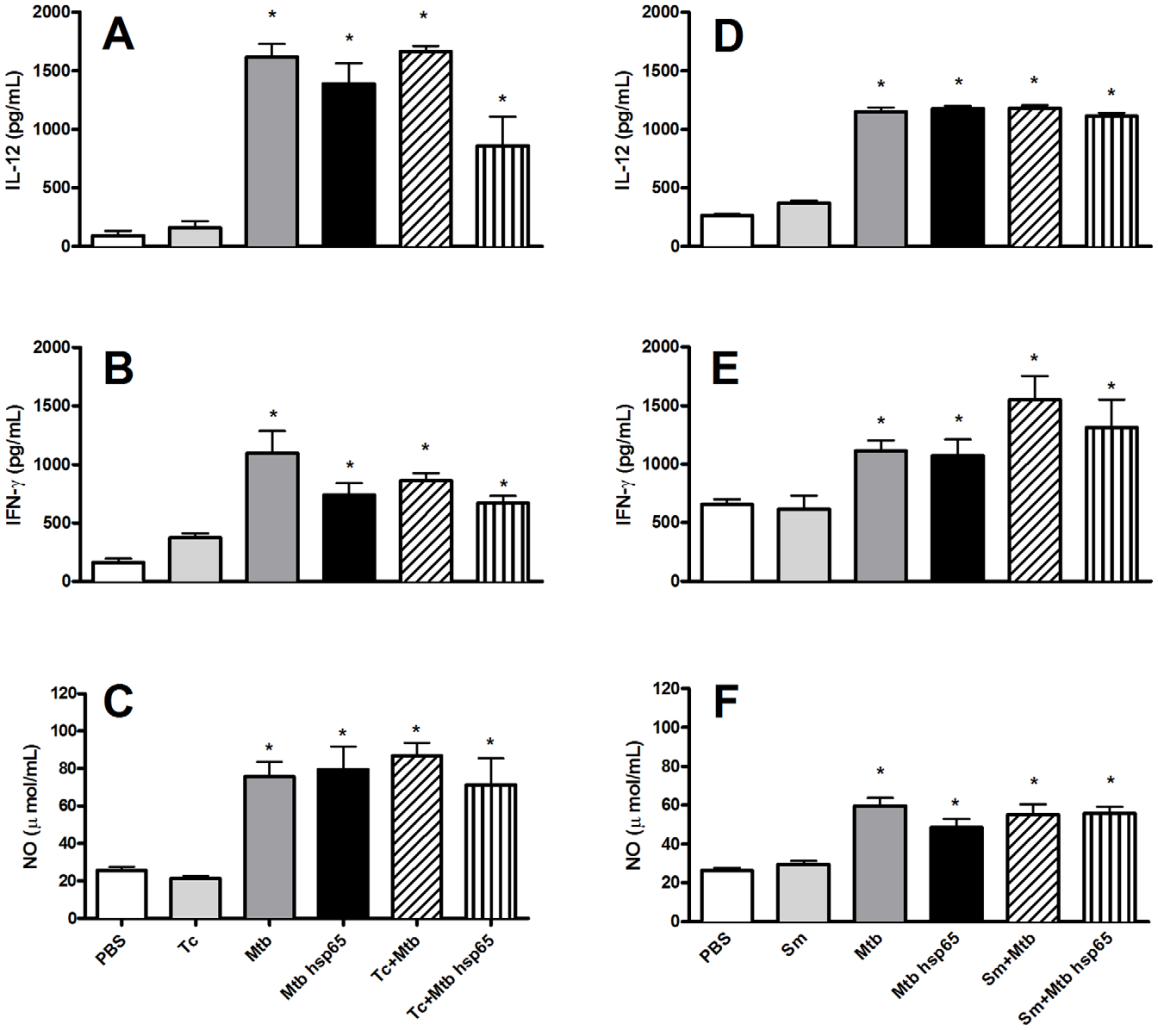
Th1 cytokine and nitrite levels in lung parenchyma from coinfected mice after treatment. Mice were infected with *T. canis* (A, B, C) or *S. mansoni* (D, E, F) and coinfected with *M. tuberculosis*. After TB establishment, mice received 4 doses of DNAhsp65 and the lungs were homogenized. Cytokine levels in supernatant were measured by ELISA and nitrite production was quantified by Griess reaction. *p<0.05 versus PBS.

As nitric oxide (NO) is an important microbicidal factor, we analyzed its production indirectly within the lung parenchyma through measurement of NO_2_^−^. Mtb-infected mice showed increased levels of NO compared to cells from control animals. Cells obtained from coinfected animals showed similar NO production levels when compared to Mtb-infected animals. DNAhsp65 treatment did not alter these high NO levels in any group (Figure 2C and F).

Since proinflammatory and Th1 cytokines play important role in the immune response against *M. tuberculosis*, we sought to investigate whether coinfection could alter cytokine production in lungs after DNAhsp65 treatment. Lung cells were obtained from animals that were either coinfected or singly Mtb-infected and treated with or without DNAhsp65. In Mtb-infected animals, IL-12 and IFN-γ levels in the lungs were increased when compared to mice receiving PBS or infected only with *T. canis* (Figure 2A and B) or *S. mansoni* (Figure 2D and E). These cytokines were present at similar levels in coinfected and Mtb-infected animals, even after DNAhsp65 treatment. Besides helminth infection are marked by a Th2 pattern, during coinfection the Th1 pattern kept elevated as seen during infection with *M. tuberculosis* only.

**Figure 3:**
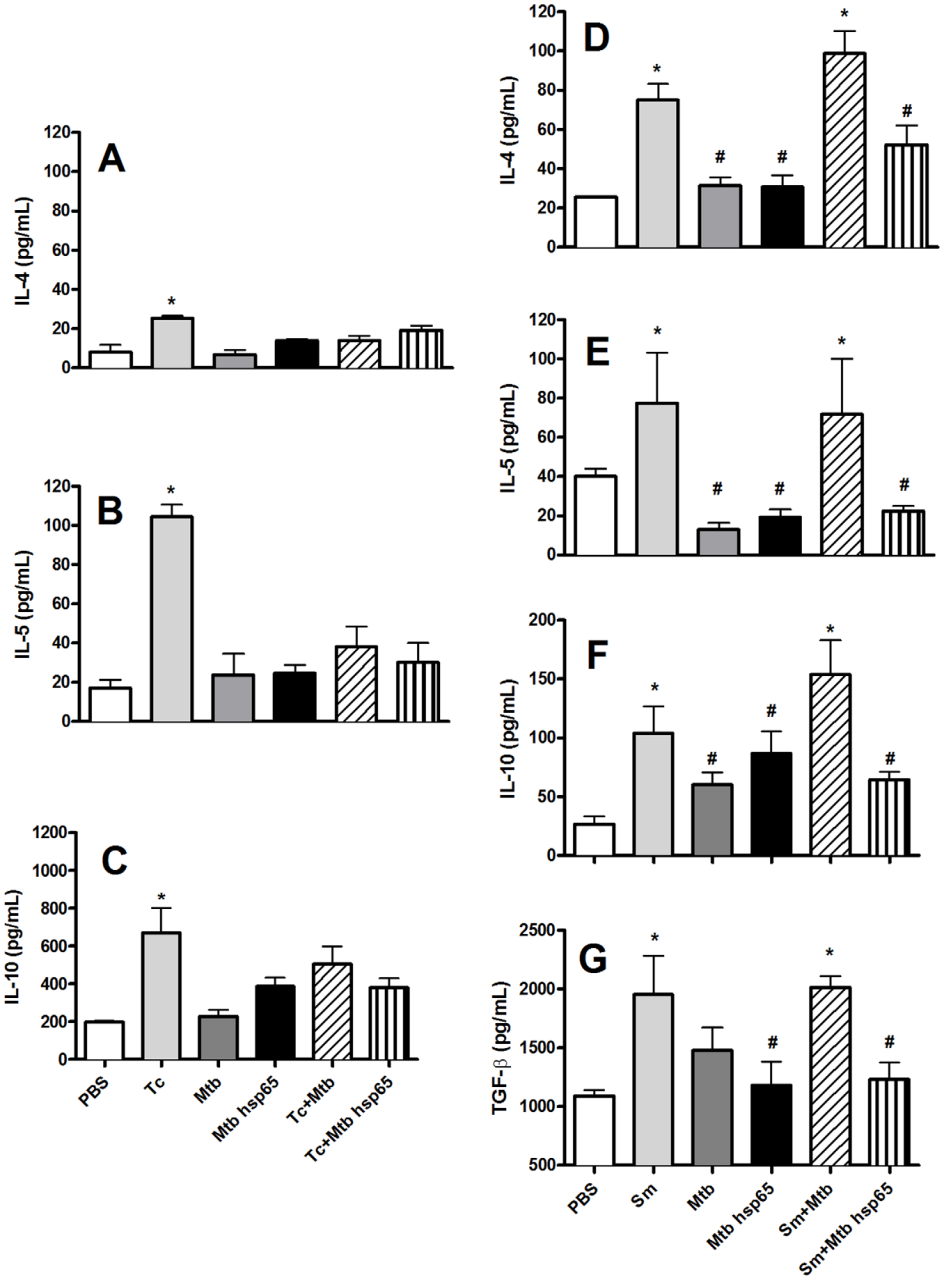
Th2 and regulatory cytokines levels released by lung cells of coinfected mice after treatment. Mice were coinfected with *T. canis* (A, B, C) or with *S. mansoni* (D, E, F, G) and received 4 doses of DNAhsp65. Ten days after the last vaccine dose, the lungs were homogenized and cytokine levels in supernatant were measured by ELISA. *p<0.05 versus PBS; ^#^p<0.05 versus Sm+Mtb or Tc+Mtb.

In an attempt to determine those elements of the immune system that respond to DNAhsp65 immunotherapy, we analyzed Th2 and regulatory cytokine profiles in lungs following DNAhsp65 treatment. Infection with *T. canis* induced elevated levels of IL-4, IL-5, and IL-10 compared to control mice (Figure 3A–C). However, in *T. canis* coinfected mice, regardless of DNAhsp65 therapy, the levels of these cytokines were reduced to control levels. Similar to *T. canis* infection, *S. mansoni* caused elevated levels of IL-4, IL-5, IL-10, and TGF-β when compared to PBS and Mtb groups (Figure 3D–G). In the *S. mansoni* coinfection scenario, these high levels of cytokine expression were maintained. Interestingly, DNAhsp65 therapy markedly reduced Th2 and regulatory cytokine levels induced by *S. mansoni*, suggesting a pathway for the protection observed in these mice.

### DNAhsp65's therapeutic effect is correlated with *in vitro* IFN-γ production

A Th1 response is a key element during the immune response against TB since it is critical in preventing progression to active disease. The presence of IFN-γ at the site of infection can circumvent the tendency of Mtb to escape phagosome maturation and facilitate control of bacterial burden [33]. To determine whether DNA immunization induced specific T cell stimulation, spleen or lung cells were obtained after coinfection and therapy and cultured in the presence of recombinant HSP65. The supernatants were collected after 48 h in cell culture and were analyzed for IFN-γ production by ELISA.

**Figure 4:**
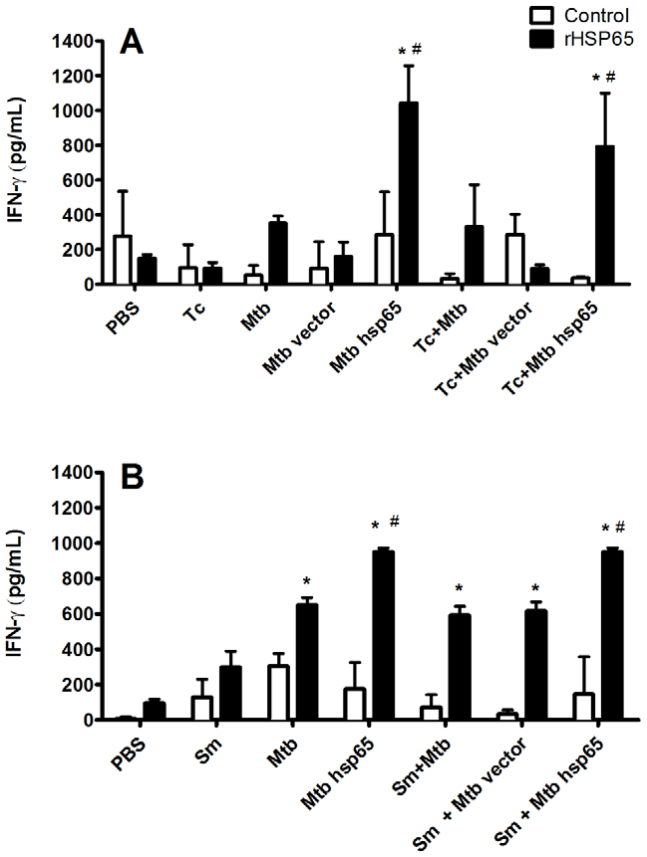
*In vitro* IFN-γ production by cells stimulated with rHSP65. After coinfection and DNAhsp65 therapy, total spleen cells (A) or lung cells (B) were stimulated *in vitro* with rHSP65 (black bars) or were left unstimulated (white bars) for 48 h. Cytokine levels in culture supernatants were determined by ELISA. *p<0.05 versus PBS; ^#^p<0.05 versus Mtb; ^&^p<0.05 versus Sm+Mtb or Tc+Mtb.

IFN-γ expression was significantly upregulated in spleen cells from infected mice receiving DNAhsp65 therapy (Figure 4A). This was observed in treated mice infected with Mtb or coinfected with *T. canis*. All other conditions produced IFN-γ levels that were not significantly different from control values. Thus, high levels of antigen-specific IFN-γ production were correlated with DNAhsp65 treatment and may account, at least in part, for the observed positive therapeutic effects.

In the *S. mansoni*-coinfection model, we analyzed HSP65-specific IFN-γ production by lung cells. As Figure 4B shows, lung cells obtained after Mtb infection produce more IFN-γ when stimulated *in vitro* with rHSP65 than lung cells from PBS and Sm groups. However, treatment with DNAhsp65 could increase these levels significantly in Mtb-infected and coinfected mice. This elevated production was not observed in untreated or vector treated mice, highlighting the specific therapeutic effects of DNA treatment in Mtb as well in coinfected groups (Figure 4).

## Discussion

According to the World Health Organization (WHO), Neglected Tropical Diseases, as helminth infections, have a major adverse impact on the health, well-being, and socioeconomic development of poverty-stricken people living in low-income countries [34], [35]. Because mycobacterial and helminth pathogens are frequently co-endemic and tend to induce opposing immune responses, we investigated the pattern of inflammatory and immune responses in coinfection models featuring pre-exposure to *T. canis* or *S. mansoni* and challenge with *M. tuberculosis* to assess effects of a therapeutic DNA vaccine that has shown efficacy in experimental TB. In our study, we showed that worm coinfection did not abrogate DNAhsp65 therapeutic effects during TB. Coinfected mice had no modulation in Th1 immune response, but present high levels of Th2 and regulatory cytokines, which was related with lung damage (data not shown). When DNA therapy was evaluated, we observed increasing in IFN-γ produced by rHsp65 recalled cells (Figure 4) and downregulation of Th2 and regulatory cytokines in the lungs of coinfected and treated mice (Figure 3). This immune modulation culminated in preserved therapeutic properties attributed to DNAhsp65 in TB/helminth coinfection models.

Our group has focused on the heat shock protein produced by *Mycobacterium leprae* (hsp65) as a vaccine antigen against several experimental pathologies including TB [20], [28], [36], diabetes [37], arthritis [38], leishmaniasis [39], and cancer in phase I clinical trials [40], [41]. The success of hsp65 vaccination in these different diseases reflects its effectiveness as an immunodominant antigen and also suggests that it produces hsp-dependent effects, such as those ascribed to other heat shock proteins [42]. The search for new vaccines and therapies against TB is due in part to the widely variable protective efficacy of BCG which ranges from 0 to 80% protection depending on the country [43], as well as the fact that BCG protects against TB in newborns, but does not prevent latent TB or reactivation in adults [44], [45]. One hypothesis regarding the variability of BCG efficacy in developing countries includes the presence of Th2-cell and Treg responses, which could be driven by co-existing helminth infections or environmental mycobacterias [46]. In 2005, Elias and coworkers showed for the first time that a helminth infection could alter the *in vivo* protective effect conferred by BCG immunization [23]. This pivotal study showed that mice previously immunized with BCG and co-infected with *S. mansoni* were more susceptible to TB, showing increased bacterial load and lung pathology. Moreover, when spleen cells from these coinfected mice were stimulated *in vitro* with PPD (purified protein derivative), they produced lower IFN-γ and nitric oxide levels.

We demonstrated previously that *T. canis* infection does not lead to increased susceptibility to pulmonary tuberculosis [22] and in the present work we show that pre-exposure to *T. canis* infection does not affect the therapeutic effects of DNAhsp65. Recently, *S mansoni* infection has been reported to increase susceptibility to TB [13], which was confirmed in the present work using a different route of TB infection. Mice coinfected with *S. mansoni* and *M. tuberculosis* presented increases in lung inflammation and CFU counts, accompanied by augmentation of Th2 cytokine release in lung tissue. In contrast, *T. canis* coinfection did not increase susceptibility to TB or alter Th1 cytokines production. These observed changes in Th1 and Th2 phenotypes may account for the differences in TB susceptibility between *S. mansoni* and *T. canis* coinfection. Despite this, the DNAhsp65 vaccine proved capable of maintaining its therapeutic properties even under TB and *S. mansoni* coinfection.

As we can observe in the Figure 3, Th2 (IL-4 and IL-5) and regulatory (IL-10 and TGF-β) cytokines were downregulated in *S. mansoni* coinfected mice treated with DNAhsp65 compared to untreated coinfected mice, correlated to CFU counts. Indeed, Th2 lymphocyte subsets have been observed in lung tissue from patients with cavitary tuberculosis, compared with Th1 subsets in non-cavitary disease, suggesting that IL-4 might be an indicator of disease severity [47] and that the Th2 environment can increase immunopathology [48]. These data suggest that the suppression of Th2 and regulatory cytokines induced by DNAhsp65 conferred the protection of the lung parenchyma observed in coinfected hosts. Mutapi and colleagues [49] suggested that Th1/Th2 dichotomy does not sufficiently explain susceptibility or resistance to schistosome infection. Hosts who produce IFN-γ/IL-4/IL-5 in association with IL-10 present high levels of infection, proposing that the regulatory component is strongest to define the severity of pathology. In our results we observed lung damage in coinfected hosts (data not shown), associated with high IFN-γ, IL-4/IL-5 and IL-10/TGF-β, but when mice were treated with DNAhsp65 the regulatory and Th2 components were inhibited and Th1 kept elevated, allowing the preservation of lung integrity. Successful TB control depends on a finely tuned system where IFN-γ provided by effector T cells confers tuberculostatic and tuberculocidal activities to macrophages [50], [51]. We suggest that the treatment downmodulated the regulatory cytokines in the lung and simultaneously increased the antigen recalled IFN-γ production, allowing to the control of TB in coinfected treated hosts.

Helminth infections can activate and expand the T regulatory cell population (Treg) in mice as well as in humans. Tregs play an important role in the suppression of Th1 functions during the immune response induced by *S. mansoni* egg antigens and can also control the Th2 response in chronic infection [52]. Elias and co-workers recently showed that peripheral blood mononuclear cells from BCG-immunized, chronically infected *S. mansoni* patients showed increased TGF-β production without an enhanced Th2 immune response when stimulated *in vitro* with PPD, indicating that this phenomenon was related to reduced immunogenicity of BCG [15]. Our data suggest that DNAhsp65 treatment inhibited the soluble factors involved in Treg–mediated immunosuppression, as indicated by the decrease in TGF-β and IL-10 production observed in treated co-infected mice. Moreover, DNAhsp65 transfection induces macrophages to produce IL-6 (Data not shown), a cytokine that decreases suppressive effects of Treg cells and allows the development of effector responses [53]. Therefore, we have shown that DNAhsp65 therapy circumvents several difficulties in coinfected hosts, probably modulating cytokines production that lead to therapeutic efficacy against TB, highlighting its potential to become a valuable tool against TB. In conclusion, this study reiterates importance of evaluating the status of hosts during vaccine development, given the high incidence of TB/helminthes coinfection.

## Supporting Information

Figure S1 Experimental protocols of coinfection and therapy. (A) BALB/c mice were orally infected with *T. canis* embryonated eggs and 18 days after infection, mice were coinfected intratracheally with *M. tuberculosis*. (B) BALB/c mice were infected with *S. mansoni* cercariae and 42 days after infection, mice were coinfected with *M. tuberculosis* intratracheally. In both protocols, DNA vaccination was initiated 30 days after TB induction on four occasions at 10-days intervals. Ten days after the last dose, mice were killed and bacterial growth, lung histology and cytokine production by lung or spleen cells were assessed.
(0.40 MB TIF)

Alternative Language Abstract S1 Translation of the abstract into Brazilian Portuguese by author FGF.
(0.03 MB DOC)
