# Supplementary figures and images for: Helminth Coinfection Does Not Affect Therapeutic Effect of a DNA Vaccine in Mice Harboring Tuberculosis
Source: PLoS Negl Trop Dis. 2010 Jun 8;4(6):e700. doi: 10.1371/journal.pntd.0000700 (PMC2882318; doi:10.1371/journal.pntd.0000700)

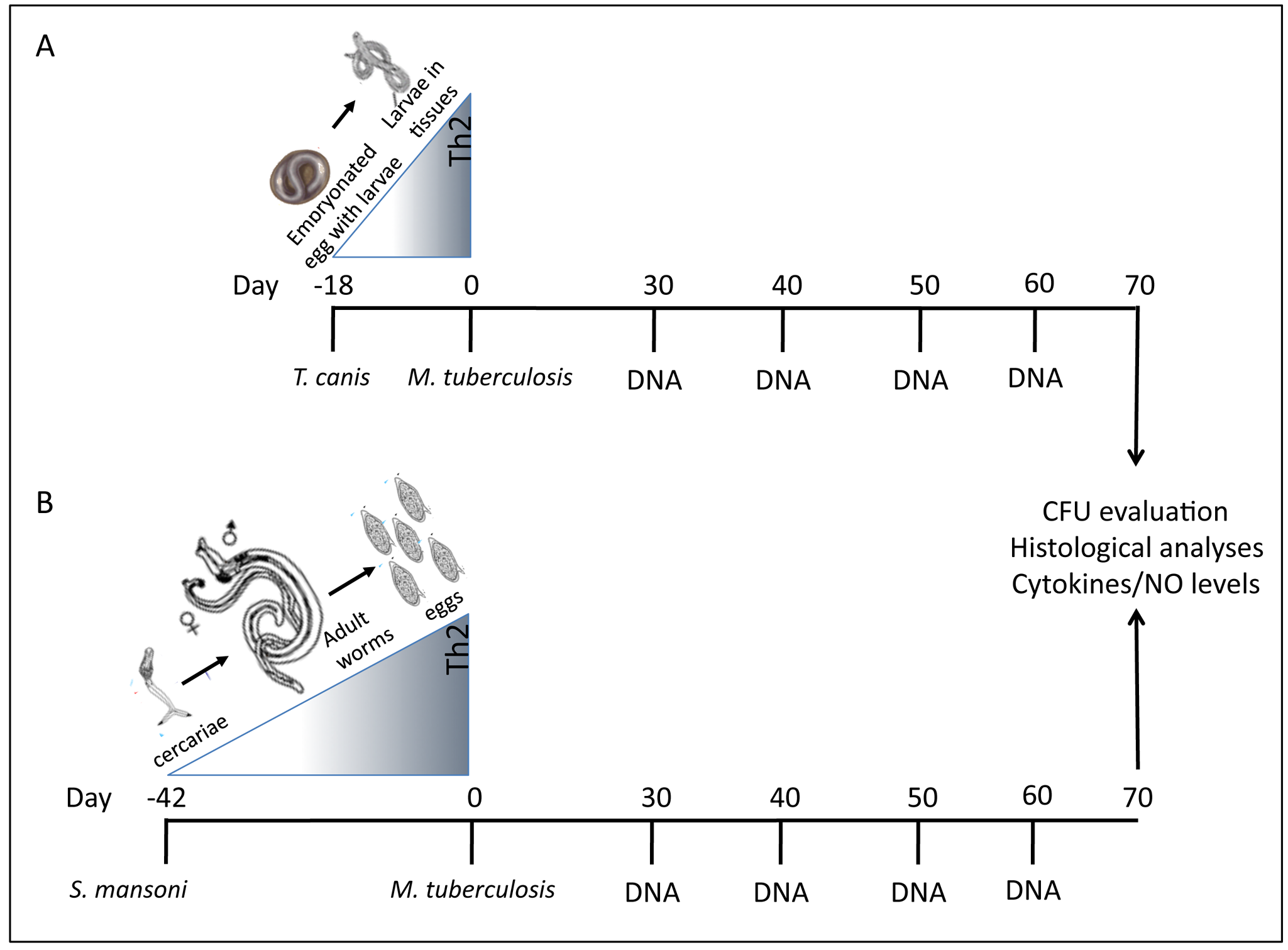

Supplement: Figure S1 — Experimental protocols of coinfection and therapy. (A) BALB/c mice were orally infected with T. canis embryonated eggs and 18 days after infection, mice were coinfected intratracheally with M. tuberculosis. (B) BALB/c mice were infected with S. mansoni cercariae and 42 days after infection, mice were coinfected with M. tuberculosis intratracheally. In both protocols, DNA vaccination was initiated 30 days after TB induction on four occasions at 10-days intervals. Ten days after the last dose, mice were killed and bacterial growth, lung histology and cytokine production by lung or spleen cells were assessed. (0.40 MB TIF) [file pntd.0000700.s001.tif]
