# Supplementary material for: Helminth Coinfection Does Not Affect Therapeutic Effect of a DNA Vaccine in Mice Harboring Tuberculosis
Source: PLoS Negl Trop Dis. 2010 Jun 8;4(6):e700. doi: 10.1371/journal.pntd.0000700 (PMC2882318; doi:10.1371/journal.pntd.0000700)
Supplement: Alternative Language Abstract S1 — Translation of the abstract into Brazilian Portuguese by author FGF. (0.03 MB DOC) [file pntd.0000700.s002.doc]

**Introdução:** Helmintíases e tuberculose (TB) coincidem geograficamente e há muito interesse em explorar como infecções por vermes alteram a resposta imune contra o bacilo e podem exigir modificações em estratégias terapêuticas. Uma vacina de DNA que codifica a proteína de choque térmico Hsp65 de *M. leprae* (DNAhsp65) tem sido utilizada na terapia da tuberculose experimental. Este estudo é centralizado no impacto da co-existência de infecção por vermes e TB nos efeitos terapêuticos da vacina DNAhsp65.

**Metodologia/Resultados:** Camundongos foram infectados com *Toxocara canis* ou com *Schistosoma mansoni*, e em seguida coinfectados com *M. tuberculosis* e tratados com DNAhsp65. Enquanto a infecção por *T. canis* não aumentou a vulnerabilidade à tuberculose pulmonar, *S. mansoni* aumentou a susceptibilidade à TB, como mostrado pelo aumento no número de bactérias recuperadas nos pulmões e baço, associado a maior produção de citocinas Th2 e reguladoras. No entanto, em camundongos coinfectados o efeito terapêutico da vacina DNAhsp65 não foi prejudicado, como indicado pela recuperação de unidades formadoras de colônia e alterações histopatológicas. Estudos *in vitro* indicaram que a produção de IFN-γ por células específicas à Hsp65 estava correlacionada com a proteção da vacina em camundongos coinfectados. Além do mais, em camundongos coinfectados com *S. mansoni* o tratamento com DNA inibiu a produção *in vivo* de TGF-β e IL-10, o que pode estar associado com a preservação da proteção contra TB.

**Conclusões:** Demonstramos que o efeito terapêutico da vacina DNAhsp65 na tuberculose experimental é mantido na presença da resposta imune Th2 induzida em infecções por helmintos.
